# Sex and survival outcomes in patients with renal cell carcinoma receiving first-line immune-based combinations

**DOI:** 10.1007/s00262-024-03719-0

**Published:** 2024-06-04

**Authors:** Lorena Incorvaia, Fernando Sabino Marques Monteiro, Francesco Massari, Se Hoon Park, Giandomenico Roviello, Ondřej Fiala, Zin W. Myint, Jakub Kucharz, Javier Molina-Cerrillo, Daniele Santini, Thomas Buttner, Alexandr Poprach, Jindrich Kopecky, Annalisa Zeppellini, Martin Pichler, Tomas Buchler, Renate Pichler, Gaetano Facchini, Andre Poisl Fay, Andrey Soares, Ray Manneh, Laura Iezzi, Zsofia Kuronya, Antonio Russo, Maria T. Bourlon, Dipen Bhuva, Jawaher Ansari, Ravindran Kanesvaran, Enrique Grande, Sebastiano Buti, Matteo Santoni

**Affiliations:** 1 Department of Precision Medicine in Medical, Surgical and Critical Care (Me.Pre.C.C.), Section of Medical Oncology, University of Palermo, 90127 Palermo, Italy; 2 Latin American Cooperative Oncology Group - LACOG, Porto Alegre, RS Brazil; 3 Oncology and Hematology Department, Hospital Sírio-Libanês, Brasilia, DF Brazil; 4 Medical Oncology, IRCCS Azienda Ospedaliero-Universitaria Di Bologna, Bologna, Italy; 5 Department of Medical and Surgical Sciences (DIMEC), University of Bologna, Bologna, Italy; 6 Division of Hematology-Oncology, Department of Medicine, Samsung Medical Center, Sungkyunkwan University School of Medicine, Seoul, 06351 Republic of Korea; 7 Department of Health Sciences, University of Florence, Florence, Italy; 8 Department of Oncology and Radiotherapeutics, Faculty of Medicine and University Hospital in Pilsen, Charles University, Alej Svobody 80, Pilsen, 304 60 Czech Republic; 9 Biomedical Center, Faculty of Medicine in Pilsen, Charles University, Alej Svobody 76, Pilsen, Czech Republic; 10 Markey Cancer Center, University of Kentucky, Lexington, KY 40536-0293 USA; 11 Department of Uro-Oncology, Maria Sklodowska-Curie National Research Institute of Oncology, Warsaw, Poland; 12 Department of Medical Oncology, University Hospital Ramón y Cajal, Madrid, Spain; 13 Department of Medical-Surgical Sciences and Biotechnology, La Sapienza University, Polo Pontino, Rome, Italy; 14 Department of Urology, University Hospital Bonn (UKB), 53127 Bonn, Germany; 15 Department of Comprehensive Cancer Care and Faculty of Medicine, Masaryk Memorial Cancer Institute and Masaryk University, Brno, Czech Republic; 16 Department of Clinical Oncology and Radiotherapy, University Hospital Hradec Kralove, Hradec Kralove, Czechia; 17 Niguarda Cancer Center, Grande Ospedale Metropolitano Niguarda, Milan, Italy; 18 Division of Oncology, Department of Internal Medicine, Medical University of Graz, 8036 Graz, Austria; 19 Department of Oncology, Second Faculty of Medicine, Charles University and University Hospital Motol, Prague, Czech Republic; 20 Department of Urology, Medical University of Innsbruck, Innsbruck, Austria; 21 Oncology Unit, “S. Maria Delle Grazie” Hospital, ASL NA2 NORD, 80078 Pozzuoli, Naples, Italy; 22 PUCRS School of Medicine, Porto Alegre, RS Brazil; 23 Latin American Cooperative Oncology Group – LACOG, Porto Alegre, Brazil; 24 Oncology and Hematology Center of Hospital Albert Einstein, Hospital Albert Einstein, Sao Paulo, Brazil; 25 Clinical Oncology, Sociedad de Oncología Y Hematología del Cesar, Valledupar, Colombia; 26 Oncology Division, Hospital ‘Maria SS. Dello Splendore’ ASL 4, Giulianova, Italy; 27 Department of Genitourinary Medical Oncology and Clinical Pharmacology, National Institute of Oncology, Budapest, Hungary; 28 Hematology and Oncology Department, Instituto Nacional de Ciencias Médicas y Nutrición Salvador Zubirán, Mexico City, Mexico; 29 Department of Medical Oncology, Army Hospital Research and Referral, New Delhi, India; 30 Medical Oncology, Tawam Hospital, Al Ain, United Arab Emirates; 31 National Cancer Centre Singapore, Singapore, Republic of Singapore; 32 Department of Medical Oncology, MD Anderson Cancer Center Madrid, Madrid, Spain; 33 Department of Medicine and Surgery, University of Parma, Via Gramsci 14, 43126 Parma, Italy; 34 Oncology Unit, University Hospital of Parma, Via Gramsci 14, 43126 Parma, Italy; 35 Oncology Unit, Macerata Hospital, Macerata, Italy

**Keywords:** ARON-1 study, Gender differences, Immunotherapy, Immune-based combinations, NCT05287464, Renal cell carcinoma

## Abstract

**Background:**

There is an ongoing debate as to whether sex could be associated with immune checkpoint inhibitor (ICI) benefit. Existing literature data reveal contradictory results, and data on first-line immune combinations are lacking.

**Method:**

This was a real-world, multicenter, international, observational study to determine the sex effects on the clinical outcomes in metastatic renal cell carcinoma (mRCC) patients treated with immuno-oncology combinations as first-line therapy.

**Results:**

A total of 1827 mRCC patients from 71 cancer centers in 21 countries were included. The median OS was 38.7 months (95% CI 32.7–44.2) in the overall study population: 40.0 months (95% CI 32.7–51.6) in males and 38.7 months (95% CI 26.4–41.0) in females (*p* = 0.202). The median OS was higher in males vs. females in patients aged 18-49y (36.9 months, 95% CI 29.0–51.6, vs. 24.8 months, 95% CI 16.8–40.4, *p* = 0.426, with + 19% of 2y-OS rate, 72% vs. 53%, *p* = 0.006), in the clear cell histology subgroup (44.2 months, 95% CI 35.8–55.7, vs. 38.7 months, 95% CI 26.0–41.0, *p* = 0.047), and in patients with sarcomatoid differentiation (34.4 months, 95% CI 26.4–59.0, vs. 15.3 months, 95% CI 8.9–41.0, *p* < 0.001). Sex female was an independent negative prognostic factor in the sarcomatoid population (HR 1.72, 95% CI 1.15 − 2.57, *p* = 0.008).

**Conclusions:**

Although the female’s innate and adaptive immunity has been observed to be more active than the male’s, women in the subgroup of clear cell histology, sarcomatoid differentiation, and those under 50 years of age showed shorter OS than males.

**Supplementary Information:**

The online version contains supplementary material available at 10.1007/s00262-024-03719-0.

## Introduction

Immunogenicity and vascularization are the main features of renal cell carcinoma (RCC) [1]. Immune-based combinations, including two immune checkpoint inhibitors (ICIs), or ICI and a tyrosine kinase inhibitor (TKI) with anti-angiogenic activity, have emerged as the standard first-line treatment [2–7].

Although immune-based therapies have clearly extended patient survival, the immunotherapy benefit and response rate are variable, and extensive efforts have been undertaken to identify robust biomarkers or clinical factors for optimal patient selection [8]. Biological factors, specific for certain individuals, have a clear effect on the variation in immunotherapy response.

Currently, there is an ongoing debate as to whether sex could be associated with ICI benefit [9]. Sex-based differences are involved in immune profiles [10], but it is unclear whether these differences play a role in immunotherapy benefit. Existing literature data reveal contradictory results. Conforti et al*.* reported in a meta-analysis of randomized clinical trials that male cancer patients treated with immune checkpoint inhibitors (ICI) derived greater efficacy than female patients [11]. Conversely, others following data found no difference in overall survival (OS) from ICIs when comparing the efficacy of these treatments between males and females [12, 13]. A retrospective analysis performed in patients with metastatic RCC (mRCC) revealed no difference between nivolumab and everolimus among the two sexes, although the small sample size limits clear conclusions [14]. The CheckMate-214 trial on nivolumab plus ipilimumab versus sunitinib in the first-line treatment of intermediate/poor-risk mRCC patients showed a wider OS advantage from ICI-combination among females (HR, 0.52; 95% CI, 0.34–0.78) compared to males (HR, 0.71; 95% CI, 0.55–0.92) [2], while inconsistent conclusions derived in mRCC population from a following meta-analysis [15].

Nevertheless, the existence of sexual dimorphism in immunological responses is supported by several observations, from the evidence of sex-associated molecular mechanisms to the reports on specific immune features potentially altering treatment responsiveness [16, 17]. In a comprehensive analysis of molecular biomarkers from The Cancer Genome Atlas (TCGA), sex-associated divergent patterns were observed in RCC. The higher mutation rate for the *PBRM1* gene was in male clear cell RCC (ccRCC) patients and higher tumor mutation burden (TMB) and cytolytic activity (CYT) of T cells in males with renal papillary cell carcinoma. In the same research, sex-based differences were also in multiple immune features, including immune checkpoints (e.g., CD28 and CD86) and immune cell populations (e.g., active CD4 + T cells) [17].

In addition to these elements, which highlight the importance of molecular profiling and immune components for the ICI response, sex imbalance includes predominance and age selectivity for women in Xp11.2 translocation RCC [18] as well as the known differences in environmental stressors and lifestyle or behavior, disparities in body mass index (BMI) and comorbidities, sex hormone modulation with updated data on hormone changes during immunotherapy, and an increased emphasis on epigenetic influence [19–22].

The ARON-1 study (ClinicalTrials.gov identifier NCT05287464) was a multicenter, international, retrospective study to collect real-world data on clinical outcomes of metastatic RCC patients treated with immuno-oncology combinations as first-line therapy [23, 24]. In the current manuscript, we present the results from the analysis focused on the impact of sex on immune-based combinations effectiveness, stratified by ICI plus ICI, or ICI plus anti-angiogenic agent.

## Patients and methods

### Study design and population

The study population included patients diagnosed at age ≥ 18 years with RCC and radiologically confirmed metastatic disease, treated from January 1st, 2016, to October 1st, 2023, in 71 cancer centers in 21 countries (Table S1). All included patients had known data on age, sex, tumor histology, prognostic risk group according to the International Metastatic Renal Cell Carcinoma Database Consortium (IMDC) criteria, previous nephrectomy, sites of metastases, type of immuno-combination, durations and response to therapy according to Response Evaluation Criteria In Solid Tumors 1.1 (RECIST 1.1) measured by investigators in each center. Pre-treatment neutrophil-to-lymphocyte ratio (NLR) and body mass index (BMI) were also collected. The NLR was recorded from the routinely performed blood cell count, as the absolute count of neutrophils divided by the absolute count of lymphocytes from peripheral blood samples collected at baseline. BMI was calculated as weight in kilograms divided by height in meters squared. Normal weight (BMI = 18.5–24.9 kg/m^2^), overweight (BMI = 25–29.9 kg/m2), and obesity (BMI ≥ 30 kg/m2) were classified based on the World Health Organization (WHO) recommendations.

Clinical data were retrospectively and locally extracted, at each participating center, from the patients' medical records. The pathological information was abstracted from pathology reports for clinical use. First-line therapy was continued until the evidence of clinical and/or radiological tumor progression, unacceptable toxicities as per clinical local practice, or death. Computed tomography (CT), magnetic resonance imaging (MRI) scans, and laboratory tests were performed following standard local procedures.

Patients with lacking the above-mentioned information were excluded from the ARON-1 study.

The study was conducted according to Good Clinical Practice (GCP) and has been designed with the ethical principles laid down in the Declaration of Helsinki on human experimentation. The study protocol was approved by the Ethical Committee of the coordinating center (Marche Region-2021-492, Study Protocol “ARON 1 Project”) and by the Institutional Review Boards of international participating centers.

### Study objectives

Our primary objective was to assess OS of metastatic patients treated with first-line immune combinations according to the patient's sex. Secondary objectives were the comparison of the tumor response [progression disease (PD), stable disease (SD), partial response (PR), complete response (CR)], objective response rate (ORR), to first-line treatment, between female and male patients. OS was calculated from the start of treatment to death for any cause. Patients without a tumor progression to the following line of treatment or death or lost at follow-up at the time of analysis were censored at their last follow-up date. Adverse events, dose reductions, and treatment interruptions were also collected.

### Statistical considerations

The comparison between subgroups was performed with the chi-square test. The best cut-off for the number of metastatic sites was calculated by ROC curve and resulted > 2. For BMI and NLR, the best cut-offs calculated by ROC curves were ≥ 25 and ≥ 4.

OS was calculated from the time of the start of first-line therapy until death. Progression-free survival (PFS) was calculated as the time from the start of first-line therapy to documented disease progression or death from any cause, whichever occurred first. Patients without disease progression or death or lost at follow-up at the time of the analysis were censored at the last follow-up visit. The analysis of OS between groups was compared using the Kaplan–Meier method and log-rank test. To identify independent prognostic factors for OS, univariate and multivariate Cox proportional hazard regression models were performed. The list of variables included sex, age, BMI, nephrectomy, sarcomatoid differentiation, IMDC group, number and type of metastatic sites.

The chi-square test was used to compare groups for categorical variables. *P* values < 0.05 were considered statistically significant. Statistical analyses were conducted using MedCalc version 19.6.4 (MedCalc Software, Broekstraat 52, 9030 Mariakerke, Belgium).

## Results

### Patients population

We included 1827 patients treated with immune combinations from the ARON-1 dataset; 1352 (74%) were males and 475 (26%) were females, with 30% of patients aged ≥ 70y. Clear cell histology was predominant (1578 patients, 86%); non-clear cell histology was in 249 patients (14%), including 97 papillary (5%), 36 chromophobe (2%), and 116 other histology subtypes (6%). In the group of non-clear cell histology, 11 patients presented Xp11.2 translocation (male-to-female ratio 4:7). Among the patients with clear cell histology, 274 patients (15%) reported sarcomatoid differentiation, [180 females (65.7%) and 94 (34.3%) males]. Lung (68%), lymph nodes (34%) and bone (33%) were the most frequent metastatic sites. The majority of patients (65%) underwent nephrectomy. Favorable, intermediate, and poor IMDC features were present in 14%, 62%, and 24% of all cases, respectively. The complete list of patients' characteristics is reported in Table 1.

**Table 1:** Patient characteristics

Characteristics	Overall No. (%)	Males No. (%)	Females No. (%)	*p* value
Total patients	1827 (100)	1352 (100)	475 (100)	–
Age (years)				
18–49	240 (13)	180 (13)	60 (13)	0.999
50–69	1046 (57)	780 (58)	266 (56)	
≥ 70	541 (30)	392 (29)	149 (31)	
Clear cell histology	1578 (86)	1170 (87)	408 (86)	0.837
Non-clear cell histology	249 (14)	182 (13)	67 (14)	0.837
Papillary	97 (5)	73 (5)	24 (5)	1.000
Chromophobe	36 (2)	25 (2)	11 (2)	1.000
Other	116 (6)	84 (6)	32 (7)	0.775
Sarcomatoid differentiation	274 (15)	180 (13)	94 (20)	0.184
Metastatic at diagnosis	1034 (57)	758 (56)	276 (58)	0.776
Previous nephrectomy	1179 (65)	881 (65)	298 (63)	0.769
No. of metastatic sites > 2	532 (29)	396 (29)	136 (29)	1.000
Site of metastasis, individual				
Lung	1251 (68)	947 (70)	304 (64)	0.368
Lymph node	614 (34)	445 (33)	169 (36)	0.656
Liver	323 (18)	225 (17)	98 (21)	0.472
Bone	603 (33)	451 (34)	152 (32)	0.764
Brain	129 (7)	88 (7)	41 (9)	0.603
IMDC Prognostic Risk Group				
Favorable	259 (14)	194 (14)	65 (14)	1.000
Intermediate	1139 (62)	852 (63)	287 (60)	0.664
Poor	429 (24)	306 (23)	123 (26)	0.623
NLR ≥ 4	533 (29)	390 (29)	143 (30)	0.877
BMI ≥ 25	1154 (63)	899 (66)	255 (54)	0.084

BMI, Body Mass Index; IQR, interquartile range; NLR, Neutrophil-to-Lymphocyte Ratio

### Survival analysis and response to first-line therapy

**Fig. 1:**
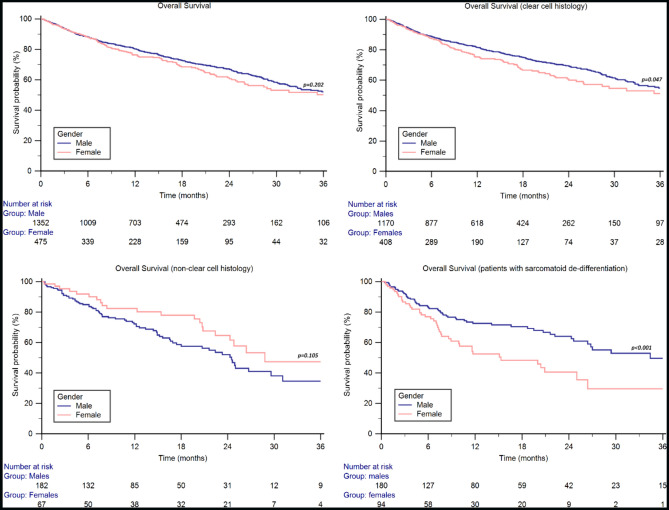
Overall Survival by gender in the ARON-1 study population and stratified by tumor histology and presence of sarcomatoid differentiation

The median OS was 38.7 months (95% CI 32.7–44.2) in the overall study population and was 40.0 months (95% CI 32.7–51.6) in males and 38.7 months (95% CI 26.4–41.0) in females (*p* = 0.202, Fig. 1). The 2y-OS rate was 67% in males and 61% in females. On the other hand, no statistically significant differences were found in terms of median PFS, which was 15.7 months (95% CI 14.3–17.6) in males and 15.7 months (95% CI 12.0–19.2, *p* = 0.259) in females (Figure S1).

In patients aged 18-49y, the median OS was 36.9 months (95% CI 29.0–51.6) in males and 24.8 months (95% CI 16.8–40.4, *p* = 0.426) in females, with + 19% of 2y-OS rate (72% vs. 53%, *p* = 0.006).

In patients aged 50-69y, the median OS was 44.2 months (95% CI 35.3–61.8) in males and NR (95% CI NR–NR, *p* = 0.533) in females, with a 2y-OS rate or 68% vs. 67%, respectively (*p* = 0.880).

In the elderly population (≥ 70y), the median OS was 31.4 months (95% CI 26.4–49.2) in males and 26.0 (95% CI 20.7–39.9, *p* = 0.525) in females, with a 2y-OS rate or 62% vs. 57%, respectively (*p* = 0.473).

The median OS was longer in males vs. females in the clear cell histology subgroup (44.2 months, 95% CI 35.8–55.7, vs. 38.7 months, 95% CI 26.0–41.0, *p* = 0.047, Fig. 1), while in the 251 patients with non-clear cell histology the median OS was higher in females, although the difference was not statistically significant (males: 24.6 months, 95% CI 20.7–31.1; females: 28.8 months, 95% CI 22.4–40.4; *p* = 0.105, Fig. 1).

In patients with sarcomatoid differentiation, the median OS was significantly longer in males (34.4 months, 95% CI 26.4–59.0 vs. 15.3 months, 95% CI 8.9–41.0, *p* < 0.001, Fig. 1), with + 20% of 1y-OS rate (71% vs. 51%) and + 24% of 2y-OS rate (64% vs. 40%).

No significant differences were found between males and females in patients with favorable (51.6 months, 95% CI 36.5–51.6 vs. NR, 95% CI NR–NR, *p* = 0.423), intermediate (40.3 months, 95% CI 31.7–55.7 vs. 35.2 months, 95% CI 26.0–44.4, *p* = 0.226) or poor-risk features (22.1 months, 95% CI 13.6–29.7, vs. 15.5 months, 95% CI 11.7–29.3, *p* = 0.730).

Furthermore, no significant differences in terms of median OS were found between males and females stratified by site of metastasis (lung: 38.9 months, 95% CI 31.7–52.2 vs. 31.6 months, 95% CI 22.4–41.0, *p* = 0.098; lymph nodes: 33.1 months, 95% CI 27.5–40.0 vs. 26.4 months, 95% CI 20.9–41.0, *p* = 0.565; bone: 26.8 months, 95% CI 22.2–30.4 vs. 26.0 months, 95% CI 17.7–29.3, *p* = 0.591; liver: 24.5 months, 95% CI 19.5–32.7 vs. 25.9 months, 95% CI 16.8–39.9, *p* = 0.746; brain: 23.6 months, 95% CI 15.4–28.2 vs. 16.8 months, 95% CI 11.7–41.0, *p* = 0.853).

Overall, 1295 patients (71%) presented 1 or 2 metastatic sites, while 532 patients (29%) reported > 2 metastatic sites. The difference between males and females was not statistically significant for both patients with 1–2 (44.2 months, 95% CI 35.3–59.0 vs. 40.2 months, 95% CI 25.9–41.0, *p* = 0.163) or with > 2 metastatic sites (29.0 months, 95% CI 25.0–40.0 vs. 28.4 months, 95% CI 24.0–39.9, *p* = 0.780).

No statistically significant differences were observed between males and females in both patients with BMI ≥ 25 kg/m^2^ (44.2 months, 95% CI 34.3–55.7 vs. NR, 95% CI NR–NR, *p* = 0.171) and BMI < 25 kg/m^2^ (31.4 months, 95% CI 27.3–40.8 vs. 31.6 months, 95% CI 25.9–40.2, *p* = 0.917) as well as in patients with NLR ≥ 4 (26.4 months, 95% CI 19.7–29.6, vs. 22.1 months, 95% CI 16.1–25.9, *p* = 0.649) or < 4 (49.2 months, 95% CI 36.9–59.6, vs. NR, 95% CI NR–NR, *p* = 0.688). The difference was slightly different when we considered patients with BMI > 30 kg/m^2^ (males: NR, 95% CI NR–NR vs. 28.8 months, 21.6–28.8, *p* = 0.081).

**Fig. 2:**
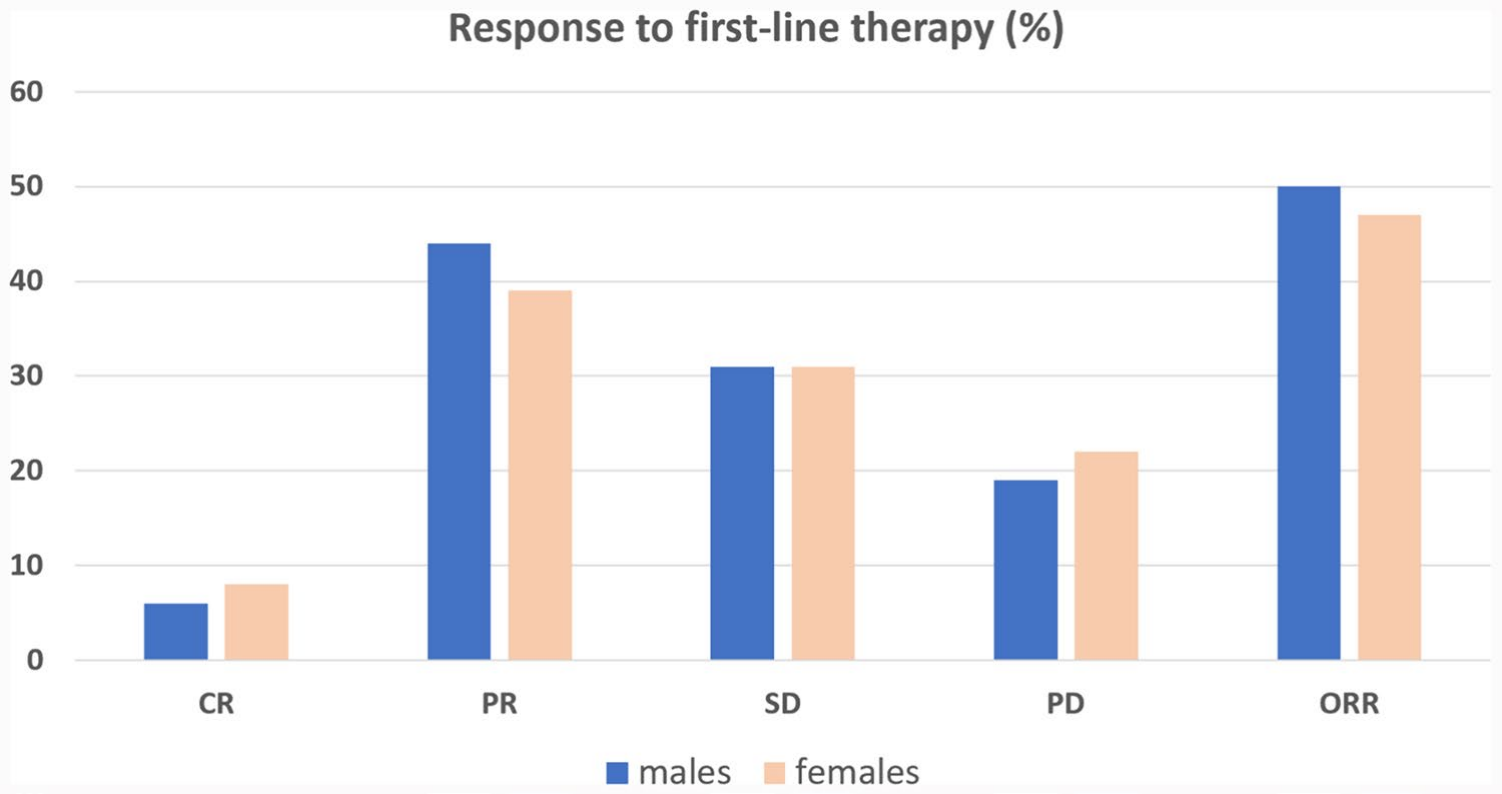
Response to therapy stratified by gender in the ARON-1 study population

Male patients showed 6% CR, 44% PR, 31% SD and 19% PD, while females reported 8% CR, 39% PR, 31% SD and 22% PD (Fig. 2). By chi-square test, no differences in terms of ORR were found in patients with clear cell (males: 52% vs. females: 49%, *p* = 0.672) or non-clear cell histology (males: 40% vs. females: 41%, *p* = 0.886).

### *IO* + *IO vs. IO* + *TKI*

IO + IO combination was the first-line therapy chosen by clinicians in 40% of males and 39% of females. Among the IO + TKI combinations, pembrolizumab plus axitinib was the most frequent, representing 45% and 44% of first-line therapies in males and females, respectively (Table 2).

**Table 2:** Overall Study Population stratified by type of first-line immune combination and gender. The percentages refer to the total number of males (1352) and females (475) included in this study

Characteristics	IO + IO		*p value*	IO + TKI		*p value*
	Males No. (%)	Females No. (%)		Males No. (%)	Females No. (%)	
Total patients	538 (40)	184 (39)	0.885	814 (60)	291 (61)	0.885
Age < 50y	65 (5)	26 (5)	1.000	115 (9)	34 (7)	0.603
Age > 70y	156 (12)	64 (13)	0.831	236 (17)	85 (18)	0.853
Clear cell histology	480 (36)	161 (34)	0.767	690 (51)	247 (52)	0.888
Sarcomatoid differentiation	101 (7)	45 (9)	0.969	79 (6)	49 (10)	0.298
Metastatic at diagnosis	333 (25)	118 (25)	1.000	425 (31)	158 (33)	0.762
Previous nephrectomy	345 (26)	114 (24)	0.744	536 (40)	184 (39)	0.885
No. of metastatic sites > 2	154 (11)	48 (10)	0.818	242 (18)	88 (19)	0.856
Site of metastasis, individual						
Lung	403 (30)	120 (25)	0.430	544 (40)	184 (39)	0.885
Lymph node	181 (13)	65 (14)	0.837	264 (20)	104 (22)	0.729
Liver	95 (7)	35 (7)	1.000	130 (10)	63 (13)	0.507
Bone	177 (13)	52 (11)	0.664	274 (20)	100 (21)	0.861
Brain	39 (3)	17 (4)	0.701	49 (4)	24 (5)	0.734
IMDC Prognostic Risk Group						
Favorable	0 (0)	0 (0)	1.000	194 (14)	65 (14)	1.000
Intermediate	390 (29)	126 (27)	0.732	462 (34)	161 (34)	1.000
Poor	148 (11)	58 (12)	0.825	158 (12)	65 (13)	0.831
NLR ≥ 4	176 (13)	55 (12)	0.831	214 (16)	88 (19)	0.578
BMI ≥ 25	374 (28)	102 (21)	0.251	525 (39)	153 (32)	0.302
First-line therapy Nivolumab plus ipilimumab Pembrolizumab plus axitinib Nivolumab plus Cabozantinib Pembrolizumab plus lenvatinib	538 (40) – – –	183 (39) – – –	0.885 – – –	– 601 (45) 153 (11) 60 (4)	– 208 (44) 53 (11) 31 (7)	– 0.887 1.000 0.353
Second-line therapy Cabozantinib Sunitinib Lenvatinib plus Everolimus Nivolumab Everolimus Clinical trials	123 (9) 57 (4) 5 (< 1) 3 (< 1) 0 (0) 18 (1)	39 (9) 22 (5) 1 (< 1) 2 (< 1) 2 (< 1) 5 (1)	-	148 (11) 39 (3) 10 (< 1) 3 (< 1) 4 (< 1) 28 (2)	67 (14) 11 (2) 0 (0) 2 (< 1) 1 (< 1) 12 (3)	–

BMI, Body Mass Index; NLR, Neutrophil-to-Lymphocyte Ratio

In the IO + IO subgroup, the median OS was 30.1 months in males (95% CI 26.7–59.0) and 26.0 months in females (95% CI 20.1–40.2, *p* = 0.325). Males showed 7% of CR, 36% PR, 35% SD, and 26% PD, while females reported 11% CR, 35% PR, 26% SD, and 28% PD.

In the IO + TKI subgroup, the median OS was 40.5 months in males (95% CI 34.3–59.6) and 40.4 months in females (95% CI 29.3–40.4, *p* = 0.402). Males showed 6% of CR, 49% PR, 32% SD, and 13% PD, while females reported 7% CR, 42% PR, 34% SD, and 17% PD.

Six hundred and two patients (33%) received second-line therapies, 32% of males and 35% of females, respectively. The complete list of second-line treatments is reported in Table 2.

### Severe adverse events, dose reductions, and treatment interruptions

Data on severe adverse events (SAEs) (Grade 3–4) were available for 1493 patients from the ARON-1 dataset and are illustrated in Table S6; SAEs were reported in 32% of males and 35% of females (Table S6).

The proportion of adverse events was higher in females than in males with BMI < 25 kg/m^2^ (female-to-male ratio, FMR: 1.6). The FMR was 3.0 for hypothyroidism and 1.5 for diarrhea.

In patients treated with the IO + IO combination, the FMR was 1.1, being 1.9 in the BMI < 25 kg/m^2^ subgroup and 1.6 for ICI interruptions. On the other hand, patients treated with IO + TKI combinations showed a FMR of 1.2. The proportion of hypothyroidism (FMR = 2.5), diarrhea (FMR = 2.0), and hypertension (FMR = 1.5) were higher in females, who were also characterized by a higher proportion of TKI interruptions (FMR = 1.2).

### Univariate and multivariate analyses

In patients with sarcomatoid differentiation, at univariate analysis, sex, age, nephrectomy status, IMDC group, bone and liver metastases were significantly associated with OS. In multivariate analysis, only the nephrectomy status did not confirm their prognostic role (Table 3).

**Table 3:** Univariate and multivariate analysis in the population of patients with sarcomatoid differentiation

Overall survival (Overall population)	Univariate Cox Regression		Multivariate Cox Regression	
	HR (95% CI )	*p* value	HR (95% CI )	*p* value
Sex (females vs. males)	1.95 (1.31 − 2.90)	**0.001**	1.72 (1.15 − 2.57)	**0.008**
Age (≥ 70y vs. < 70y)	1.52 (1.01 − 2.28)	**0.045**	1.64 (1.07 − 2.51)	**0.022**
BMI (> 25 vs. ≤ 25)	0.93 (0.63 − 1.38)	0.732		
Nephrectomy (yes vs. no)	0.48 (0.31 − 0.75)	**0.001**	0.74 (0.47 − 1.17)	0.199
Histology (nccRCC vs. ccRCC)	1.04 (0.81 − 1.32)	0.773		
IMDC group (poor vs. intermediate)	2.13 (1.46 − 3.10)	** < 0.001**	2.13 (1.42 − 3.19)	** < 0.001**
Number of metastatic sites (> 2 vs ≤ 2)	1.19 (0.80 − 1.78)	0.382		
Lung metastases (yes vs. no)	0.87 (0.57 − 1.33)	0.519		
Lymph node metastases (yes vs. no)	1.09 (0.84 − 1.41)	0.505		
Bone metastases (yes vs. no)	1.75 (1.18–2.60)	**0.005**	1.62 (1.08–2.43)	**0.021**
Liver metastases (yes vs. no)	1.82 (1.18 − 2.80)	**0.006**	1.78 (1.15–2.75)	**0.010**
Brain metastases (yes vs. no)	1.61 (0.91 − 2.83)	0.101		

BMI = Body Mass Index; ccRCC = clear cell Renal Cell Carcinoma; IMDC = International Metastatic RCC Database Consortium; nccRCC = non-clear cell Renal Cell Carcinoma

In the clear cell histology subgroup, at univariate analysis, sex, age, BMI, nephrectomy status, sarcomatoid differentiation, IMDC group, number of metastatic sites, lymph node, bone, liver and brain metastases were significantly associated with OS. In multivariate analysis, age, nephrectomy status, sarcomatoid differentiation, IMDC group, bone, liver and brain metastases confirm their prognostic role (Table S2).

In the non-clear cell histology subgroup, at univariate analysis, sex, age, BMI, nephrectomy status, sarcomatoid differentiation, IMDC group, number of metastatic sites, lymph node, bone, liver and brain metastases were significantly associated with OS. In multivariate analysis, age, nephrectomy status, sarcomatoid differentiation, IMDC group, bone, liver and brain metastases confirm their prognostic role (Table S3).

Univariate and multivariate analyses in the overall population and patients under 50 years of age are presented in Tables S4 and S5, respectively.

## Discussion

Sex is a known factor that influences cancer occurrence, progression, and prognosis [25, 26]. Sex differences are observed in the innate and adaptive immune escape mechanisms. Female seems to have a more efficient and stronger immune response than males, representing most cases of autoimmune disease, just as women infected with HIV have a lower viral load than men [27, 28]. The underlying biological, hormonal, and metabolic mechanisms explaining these findings might be also involved in sex-specific differences in immunotherapy response.

Previous research showed that mRCC female patients treated with ICI in the US in 2015–2016, i.e., before approval of immune-based combinations, had an increased risk of death than male patients (HR: 1.12, *p* = 0.004) [29]. Otherwise, a meta-analysis that included three trials with immune checkpoint inhibitor combination therapies in the first-line setting and one trial with ICI in the second-line did not demonstrate any difference in survival between males and females [30]. To date, real-world data on the possible association between sex and oncological outcomes in patients treated with first-line immune-based combinations are lacking. With this goal, we performed a sub-analysis of the international, real-world, ARON-1 study, which included 1827 patients treated with IO + IO vs. IO + TKI in 71 cancer centers in 21 countries.

For the entire cohort, we did not demonstrate the difference in OS and ORR between the sexes. Interestingly, we observed that among patients with clear cell histology, sarcomatoid differentiation, or < 50 years of age, the females showed lower OS than males. Specifically, the 2y-OS rate was 19% higher for male patients aged 18-49y (males vs. females, 72% vs. 53%, *p* = 0.006). Our results do not confirm previous data on localized disease showing a reduced risk of death in female RCC patients with < 59 years [31]. Recent OS results from the third prespecified interim analysis of Phase 3 KEYNOTE-564 study showed OS benefit for the adjuvant pembrolizumab vs. placebo for the treatment of clear cell RCC. Interestingly, greater OS benefit was for males [HR (95% CI), female vs. male: HR 1.08 (0.57–2.04) vs. 0.50 (0.33–0.75), respectively] and patients under 65 years of age [32].

At multivariate analyses, the negative impact of female sex on OS was maintained only in patients with sarcomatoid histology. These data are consistent with our previous work including patients with sarcomatoid histology treated with cabozantinib in advanced treatment lines [33] and suggest sex female as a prognostic negative factor irrespective to treatment administered.

One reason for these findings could be the sex hormone-associated immunomodulation of the RCC tumor microenvironment (TME) [34, 35]. Recent research using single-cell RNA sequencing data showed that RCC TME of males had a higher level of CD8 + T-cell infiltration than females, but were mostly exhausted CD8 + T-cells, where the exhausted state was induced by androgen [36]. This androgen-mediated immune dysfunction in male RCC patients could play a key role in the ICI response; the PD-1/PD-L1 inhibitors, through the hormonal effects on the PD-1/PD-L1 pathway and the reinvigoration of T-cell activities, could make the tumors markedly more responsive to immunotherapy [36].

Sex hormones can also shape the interaction between the immune system and genes, extending their influence at the epigenetic level [37]. In RCC, sex-specific mutation spectra emerged and involved mutations of X chromosome encoded genes, such as *KDM5C*, and chromatin remodeling genes with epigenetic effects, such as *BAP1*. A mutation sequencing study on clear cell RCC identified a significantly increased mutation rate of *KDM5C* in male and *BAP1* in female patients [38]. Interestingly, mutations of *BAP1* gene were previously associated with poorer prognosis in RCC, highlighting potential implications of genetic and epigenetic events in the sex-related disparities of the clinical outcome [30].

Sex hormones, in particular androgens, seem to have a critical influence also on the gut microbiota composition [16]. In mouse model, the sex differences in gut microbiota started with puberty, and upon castration, the gut microbiota of the male was similar to the female [39]. Early life microbial exposures determined sex hormone levels and the transfer of gut microbiota from male to female resulted in increased female testosterone, reduced autoimmunity, and metabolic variations [40]. Although hormones and genes are the most well-characterized factors mediating the sex differences in immune responses, lifestyle and other environmental variables can also contribute to differential modulation of the immune system between the sexes. A sex dimorphism is reported in many health-related behaviors, such as diet, physical activities, or alcohol consumption. These factors, which further affect the patient’s immune status and immune surveillance, are extremely difficult to measure and reflect the complex interactions among environmental, biological and immunological elements, that require a deeper understanding and future efforts to explain sex and gender disparities in anti-cancer immune response and cancer immunotherapy efficacy [41]. The type of systemic treatment in our study cohort follows the available standard of care for mRCC, with the majority of patients having received IO + TKI equally between the genders. There was no difference in OS according to the type of immune-based combination; however, numerically, the ORR was higher in female patients treated with IO + IO and in male patients treated with IO + TKI.

According to previous data on different cancer types treated with ICI, showing that female patients have an increased risk of severe adverse events than male patients [42], the proportion of severe adverse events such as hypothyroidism, diarrhea, and hypertension, as well as treatment interruptions, were higher in women treated with IO + TKI. The greater risk for autoimmunity and severe side effects appear paradoxically conflicting with the less favorable immunotherapeutic response. The highest incidence in females of autoimmune diseases and SAEs may be partly explained by the localization of specific genes and micro-RNAs to the X-chromosome and by escape from physiological X-chromosome inactivation in immune cells in women [43]. At the same time, sex hormone-dependent differences affect the immune cell numbers, composition and ratio. Thus, younger females, compared to older females and males, tend to have cold and immune-infiltrated tumors, but with higher expression of inhibitory immune checkpoints, higher density of immune-suppressive cells including regulatory T cells (T-Reg), and tumor neoantigens less visible to the immune system, ultimately less responsive when stimulated by ICI [16, 44, 45].

It is important to underline that, in our study, only 26% of patients were female. This imbalance in gender representation is similar to that reported by the Checkmate-214 trial and it is in line with less than 30% of female patients included in the Keynote-426, Checkmate-9ER, and CLEAR trials [5–7]. Despite the incidence ratio of RCC in males to females of 2:1 [46] and the implemented cancer clinical trials in the last 20 years, the issue of under-representation of female patients remains to be addressed [47]. Considering the gender differences in the immune system, behaviors, and lifestyle, as well as the heterogeneity of efficacy and outcomes with ICI treatment [48], specific measures such as stratifying patients by gender should be promptly applied in clinical trials [49].

The current study has several strengths, including the large sample size. To our knowledge, it is the first real-world study that investigates the influence of sex on first-line immune combinations and that showed the female sex as a negative prognostic factor in patients with sarcomatoid histology.

We acknowledge some limitations of our study. First, the retrospective data analysis and, consequently, the lack of a central radiological review to confirm response and/or progression, and to assess the ORR. Second, data on other environmental factors or molecular and epigenetic events, still not fully understood, could act as modifiers on TME, further influencing the response to immune-based combinations. A further understanding of sex differences in epidemiology, biology, and treatment outcomes will help to personalize the therapeutic choices for the mRCC patients.

## Conclusion

Although the female’s innate and adaptive immunity has been observed to be more active than the male’s, our real-world evidence on mRCC patients treated with immune-based combinations in first-line setting did not demonstrate a difference in OS and ORR in the overall population. However, women in the subgroup of clear cell histology, sarcomatoid differentiation, and those under 50 years of age showed shorter OS than males. Female sex was an independent negative prognostic factor in the sarcomatoid histology population. A further understanding is required to better clinically address these differences.

### Supplementary Information

Below is the link to the electronic supplementary material.

Supplementary file1 (DOCX 136 kb)
