# Supplementary material for: Sex and survival outcomes in patients with renal cell carcinoma receiving first-line immune-based combinations
Source: Cancer Immunol Immunother. 2024 Jun 4;73(8):142. doi: 10.1007/s00262-024-03719-0 (PMC11150359; doi:10.1007/s00262-024-03719-0)
Supplement: Supplementary file 1 — Supplementary file1 (DOCX 136 kb) [file 262_2024_3719_MOESM1_ESM.docx]

**Supplementary Materials**

**Table S1.** List of Countries participating to the ARON-1 study.

| **List of Countries** | | |
| --- | --- | --- |
| Austria | Belgium | Brazil |
| Colombia | Czech Republic | Finland |
| Germany | Greece | Hungary |
| India | Italy | Korea |
| Mexico | Poland | Portugal |
| Romania | Singapore | Spain |
| United Arab Emirates | United Kingdom | United States |

**Table S2.** Univariate and multivariate analysis in the clear-cell histology population.

| **Overall Survival (Overall population)** | **Univariate Cox Regression** | | **Multivariate Cox Regression** | |
| --- | --- | --- | --- | --- |
|  | **HR (95%CI)** | ***p-value*** | **HR (95%CI)** | ***p-value*** |
| Sex (females vs males) | 1.24 (1.00−1.52) | **0.048** | 1.08 (0.87−1.35) | 0.493 |
| Age (≥70y vs <70y) | 1.35 (1.11−1.64) | **0.003** | 1.40 (1.14−1.72) | **0.002** |
| BMI (>25 vs ≤25) | 0.72 (0.60−0.88) | **<0.001** | 0.85 (0.69−1.04) | 0.105 |
| Nephrectomy (yes vs no) | 0.45 (0.37−0.54) | **<0.001** | 0.62 (0.50−0.77) | **<0.001** |
| Sarcomatoid differentiation (yes vs no) | 1.69 (1.34−2.13) | **<0.001** | 1.75 (1.37−2.24) | **<0.001** |
| IMDC group (poor vs intermediate) | 2.05 (1.80−2.39) | **<0.001** | 1.72 (1.45−2.04) | **<0.001** |
| Number of metastatic sites (>2 vs ≤2) | 1.53 (1.26−1.86) | **<0.001** | 0.97 (0.77−1.21) | 0.781 |
| Lung metastases (yes vs no) | 1.21 (0.98−1.51) | 0.083 |  |  |
| Lymph node metastases (yes vs no) | 1.32 (1.15−1.51) | **<0.001** | 1.16 (0.98-1.37) | 0.080 |
| Bone metastases (yes vs no) | 1.83 (1.52-2.22) | **<0.001** | 1.53 (1.24-1.88) | **<0.001** |
| Liver metastases (yes vs no) | 1.85 (1.49−2.90) | **<0.001** | 1.44 (1.13-1.83) | **0.003** |
| Brain metastases (yes vs no) | 2.16 (1.63−2.86) | **<0.001** | 1.81 (1.32-2.48) | **<0.001** |
| BMI = Body Mass Index; ccRCC = clear cell Renal Cell Carcinoma; IMDC = International Metastatic RCC Database Consortium; nccRCC = non-clear cell Renal Cell Carcinoma. | | | | |

**Table S3.** Univariate and multivariate analysis in the non-clear-cell histology population.

| **Overall Survival (Overall population)** | **Univariate Cox Regression** | | **Multivariate Cox Regression** | |
| --- | --- | --- | --- | --- |
|  | **HR (95%CI)** | ***p-value*** | **HR (95%CI)** | ***p-value*** |
| Sex (females vs males) | 0.67 (0.42−1.09) | 0.108 |  |  |
| Age (≥70y vs <70y) | 1.66 (1.09−2.53) | **0.018** | 2.00 (1.25−3.21) | **0.004** |
| BMI (>25 vs ≤25) | 0.71 (0.48−1.07) | 0.101 |  |  |
| Nephrectomy (yes vs no) | 0.37 (0.24−0.58) | **<0.001** | 0.42 (0.26−0.69) | **<0.001** |
| IMDC group (poor vs intermediate) | 1.98 (1.38−2.83) | **<0.001** | 1.49 (0.97−2.29) | 0.068 |
| Number of metastatic sites (>2 vs ≤2) | 1.09 (0.71−1.67) | 0.690 |  |  |
| Lung metastases (yes vs no) | 0.97 (0.64−1.45) | 0.873 |  |  |
| Lymph node metastases (yes vs no) | 0.82 (0.54−1.24) | 0.359 |  |  |
| Bone metastases (yes vs no) | 1.91 (1.28-2.86) | **0.002** | 1.85 (1.17-2.91) | **0.008** |
| Liver metastases (yes vs no) | 1.65 (1.07−2.56) | **0.023** | 1.38 (0.86-2.22) | 0.186 |
| Brain metastases (yes vs no) | 0.97 (0.42−2.23) | 0.937 |  |  |
| BMI = Body Mass Index; ccRCC = clear cell Renal Cell Carcinoma; IMDC = International Metastatic RCC Database Consortium; nccRCC = non-clear cell Renal Cell Carcinoma. | | | | |

**Table S4.** Univariate and multivariate analysis in the overall study population.

| **Overall Survival (Overall population)** | **Univariate Cox Regression** | | **Multivariate Cox Regression** | |
| --- | --- | --- | --- | --- |
|  | **HR (95%CI)** | ***p-value*** | **HR (95%CI)** | ***p-value*** |
| Sex (females vs males) | 1.13 (0.94−1.37) | 0.202 |  |  |
| Age (≥70y vs <70y) | 1.38 (1.16−1.65) | **<0.001** | 1.38 (1.14−1.67) | **<0.001** |
| BMI (>25 vs ≤25) | 0.71 (0.60−0.84) | **<0.001** | 0.80 (0.66−0.96) | **0.016** |
| Nephrectomy (yes vs no) | 0.43 (0.36−0.51) | **0.002** | 0.59 (0.49−0.72) | **<0.001** |
| Histology (nccRCC vs ccRCC) | 1.50 (1.20−1.88) | **<0.001** | 1.29 (0.99−1.67) | 0.052 |
| Sarcomatoid differentiation (yes vs no) | 1.60 (1.29−1.99) | **<0.001** | 1.63 (1.29−2.04) | **<0.001** |
| IMDC group (poor vs intermediate) | 2.04 (1.77−2.35) | **<0.001** | 1.65 (1.40−1.94) | **<0.001** |
| Number of metastatic sites (>2 vs ≤2) | 1.46 (1.22−1.74) | **<0.001** | 0.99 (0.80−1.21) | 0.918 |
| Lung metastases (yes vs no) | 1.11 (0.92−1.34) | 0.284 |  |  |
| Lymph node metastases (yes vs no) | 1.27 (1.11−1.44) | **<0.001** | 1.09 (0.92-1.29) | 0.309 |
| Bone metastases (yes vs no) | 1.86 (1.57-2.21) | **<0.001** | 1.53 (1.26-1.85) | **<0.001** |
| Liver metastases (yes vs no) | 1.85 (1.52−2.24) | **<0.001** | 1.43 (1.15-1.77) | **0.001** |
| Brain metastases (yes vs no) | 1.96 (1.50−2.56) | **<0.001** | 1.71 (1.28-2.29) | **<0.001** |
| BMI = Body Mass Index; ccRCC = clear cell Renal Cell Carcinoma; IMDC = International Metastatic RCC Database Consortium; nccRCC = non-clear cell Renal Cell Carcinoma. | | | | |

**Table S5.** Univariate and multivariate analysis in the in patients aged 18-49 years.

| **Overall Survival (Overall population)** | **Univariate Cox Regression** | | **Multivariate Cox Regression** | |
| --- | --- | --- | --- | --- |
|  | **HR (95%CI)** | ***p-value*** | **HR (95%CI)** | ***p-value*** |
| Sex (females vs males) | 1.19 (0.73−1.93) | 0.489 |  |  |
| BMI (>25 vs ≤25) | 0.66 (0.43−1.00) | 0.051 |  |  |
| Nephrectomy (yes vs no) | 0.39 (0.25−0.60) | **<0.001** | 0.55 (0.33−0.92) | **0.022** |
| Histology (nccRCC vs ccRCC) | 1.06 (0.85−1.34) | 0.582 |  |  |
| Sarcomatoid differentiation (yes vs no) | 1.83 (1.03−3.27) | **0.039** | 2.27 (1.23−4.18) | **0.008** |
| IMDC group (poor vs intermediate) | 2.62 (1.74−3.93) | **<0.001** | 1.88 (1.17−3.00) | **0.009** |
| Number of metastatic sites (>2 vs ≤2) | 1.52 (0.99−2.34) | 0.054 |  |  |
| Lung metastases (yes vs no) | 1.64 (1.01−2.66) | **0.046** | 1.40 (0.82−2.40) | 0.218 |
| Lymph node metastases (yes vs no) | 1.34 (0.86−2.08) | 0.191 |  |  |
| Bone metastases (yes vs no) | 2.02 (1.31-3.10) | **<0.001** | 1.46 (0.90-2.36) | 0.127 |
| Liver metastases (yes vs no) | 2.75 (1.78−4.24) | **<0.001** | 2.17 (1.35-3.48) | **0.001** |
| Brain metastases (yes vs no) | 1.78 (0.99−3.23) | 0.055 |  |  |
| BMI = Body Mass Index; ccRCC = clear cell Renal Cell Carcinoma; IMDC = International Metastatic RCC Database Consortium; nccRCC = non-clear cell Renal Cell Carcinoma. | | | | |

**Table S6** Severe adverse events and drug interruptions or dose reductions.

| **Characteristics** | **Overall**  **1493 (%)** | **Males**  **1109 (%)** | **Females**  **384 (%)** | **IO + IO** | | **IO + TKI** | |
| --- | --- | --- | --- | --- | --- | --- | --- |
|  |  |  |  | **Males**  **435 (%)** | **Females**  **157 (%)** | **Males**  **674 (%)** | **Females**  **227 (%)** |
| **Severe adverse events** | 32 | 32 | 35 | 28 | 32 | 33 | 38 |
| **Severe adverse events (BMI≥25)** | 21 | 21 | 22 | 18 | 19 | 22 | 23 |
| **Severe adverse events (BMI<25)** | 11 | 9 | 14 | 7 | 13 | 11 | 15 |
| **G3-G4 Hypothyroidism** | 3 | 2 | 6 | 3 | 4 | 2 | 5 |
| **G3-G4 Diarrhea** | 7 | 6 | 9 | 7 | 9 | 5 | 10 |
| **G3-G4 Fatigue** | 7 | 7 | 7 | 5 | 4 | 8 | 9 |
| **G3-G4 Hand-Foot Syndrome** | 2 | 2 | 2 | 0 | 0 | 4 | 3 |
| **G3-G4 Hypertension** | 4 | 4 | 5 | 1 | <1 | 6 | 9 |
| **TKI dose reductions** | 45 | 42 | 52 | - | - | 42 | 52 |
| **TKI interruptions due to SAEs** | 23 | 24 | 21 | - | - | 24 | 21 |
| **ICI interruptions due to SAEs** | 18 | 17 | 21 | 23 | 36 | 15 | 17 |

**Figure S1.**

**
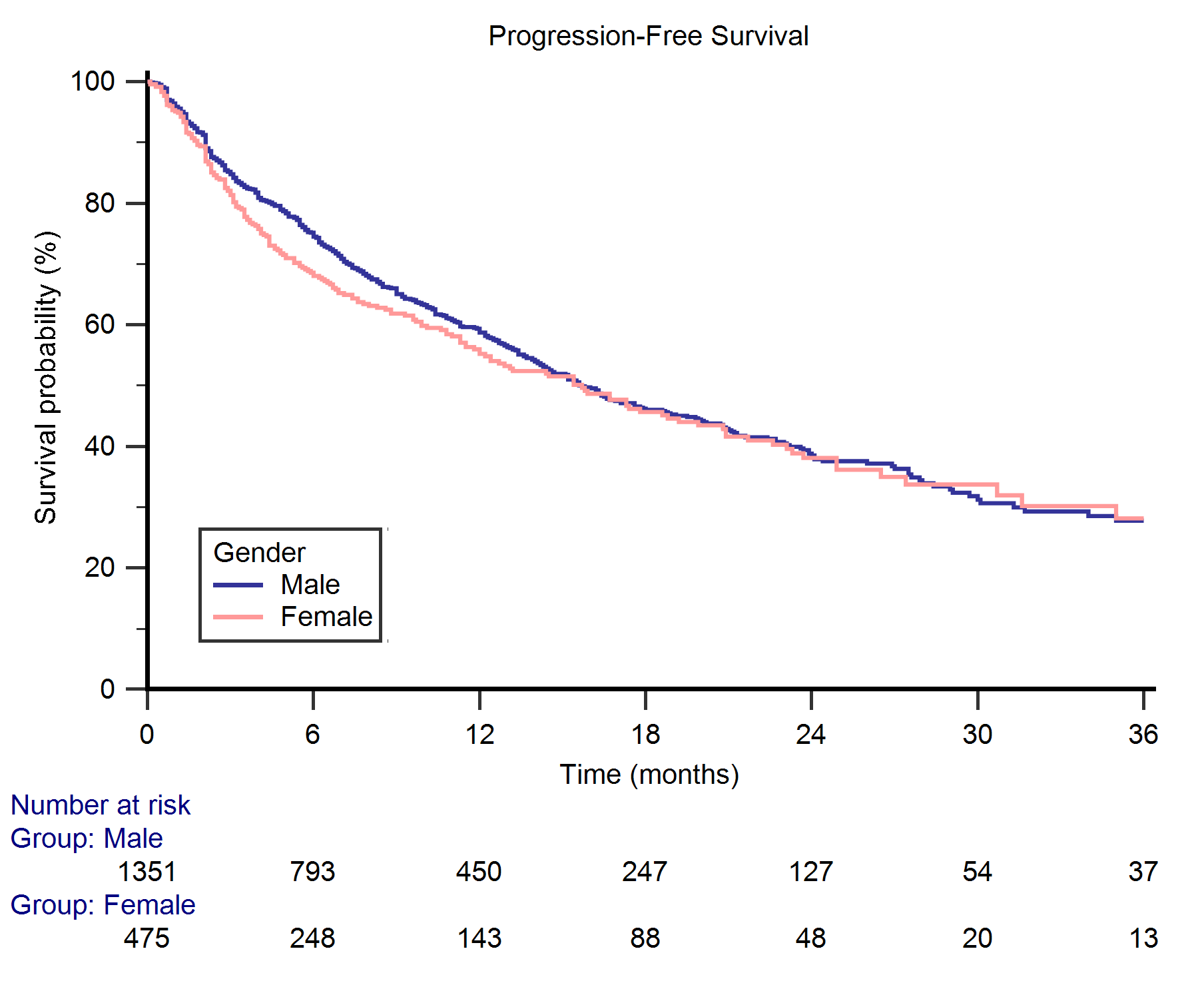
**
